# Prevalence of Pulmonary Tuberculosis and Rifampicin Resistance Among Patients Attending Adama Hospital Medical College

**DOI:** 10.1155/ijm/1475396

**Published:** 2025-02-12

**Authors:** Hawi Kumbi, Alegntaw Abate, Abdela Kedir, Tesfaye Chala, Milkesa Gemechu, Alemayehu Garedew, Musa Ali

**Affiliations:** ^1^Adama Hospital Medical College, Adama, Oromia, Ethiopia; ^2^Department of Medical Laboratory Science, College of Health Sciences, Oda Bultum University, Chiro, Oromia, Ethiopia; ^3^School of Medical Laboratory Science, College of Medicine and Health Sciences, Hawassa University, Awasa, Sidama, Ethiopia

**Keywords:** Adama, Ethiopia, GeneXpert, *Mycobacterium tuberculosis*, Oromia, prevalence, rifampicin, tuberculosis

## Abstract

**Background:** Tuberculosis (TB) is among the leading causes of morbidity and mortality in resource-limited countries. The burden of TB varies from country to country, depending on the country's condition and the effort made to prevent its transmission. The magnitude of pulmonary TB and drug resistance in eastern Ethiopia is mainly unknown due to limited information.

**Objective:** This study aimed to determine the prevalence of pulmonary TB and rifampicin-resistant *Mycobacterium tuberculosis* and factors associated with pulmonary TB.

**Methods:** A hospital-based prospective cross-sectional study was conducted among 424 presumptive TB patients who attended Adama Hospital Medical College from January 10, 2023, to November 10, 2023. Sputum (gastric lavage for children) was collected and diagnosed using the Ziehl–Neelsen (ZN) staining method and GeneXpert. A structured questionnaire was used to collect sociodemographic and clinical data. SPSS Version 20 computer software was used for data analysis. A variable with a *p* value less than 0.05 was considered statistically significant.

**Results:** The prevalence of the ZN staining method and GeneXpert-confirmed TB was 160 (37.7%), 95% CI: 33–42.7 and 189 (44.6%), 95% CI: 39.8–49.5, respectively. Of the study participants, nine (2.1%) were infected with rifampicin-resistant *M. tuberculosis*. Out of the 189 confirmed TB cases, 4.7% were infected with rifampicin-resistant gene-positive *M. tuberculosis*. Gender—male (AOR = 1.47 [0.95–2.26], *p*=0.081), history of contact with TB patient (AOR = 7.19 [2.55–20.25], *p* < 0.001), previously treated TB patients (AOR = 3.11 [1.49–6.50], *p*=0.003), and smoking cigarette (AOR = 14.8 [1.88–117], *p*=0.010) were significantly associated with GeneXpert-confirmed pulmonary TB.

**Conclusion:** The prevalence of pulmonary TB was high, with a moderate proportion of rifampicin-resistant gene-carrying *M. tuberculosis* in the study area. Being male, having a history of contact with TB patients, having a history of infection with TB, and smoking cigarettes were significant predictors of pulmonary TB.

## 1. Background

Tuberculosis (TB) is caused by *Mycobacterium tuberculosis*, which is spread from person to person via respiratory route. *M. tuberculosis* mainly affects the lungs and causes pulmonary TB with the possibility of dissemination to other body parts [1]. When people with active pulmonary TB cough, sneeze, talk, sing, or spit, they expel infectious aerosol droplets into the air, putting others in the area at risk of infection. A sneeze can release up to 40,000 droplets [2, 3]. Inhalation of droplets containing fewer than 10 bacteria can initiate infection [3, 4].

Worldwide, TB remains a leading cause of morbidity and mortality. Globally, TB kills about three people every minute [5]. Globally in 2023, an estimated 10.8 million people fell ill with new TB cases. At the regional level, trends in TB incidence rates vary. The rate continued to increase in 2023 in two WHO regions: the Americas and the Western Pacific. After 2 years of increases, it fell slightly in the WHO Eastern Mediterranean and South-East Asia regions. From a geographical perspective, the majority of new TB cases in 2023 were concentrated in the WHO regions of southeast Asia (45%), Africa (24%), and the Western Pacific (17%), while smaller percentages were observed in the eastern Mediterranean (8.6%), the Americas (3.2%), and Europe (2.1%) [6]. The proportion of TB patients with MDR/RR-MTB in 2021 was 3.6% among new cases and 18% among those previously treated [7].

Factors contributing to increased TB incidence and mortality in developing countries include poverty, poor nutrition, overcrowded living conditions, poor ventilation, and poor hygiene [8, 9]. The WHO TB End Strategies focus on assessing the TB epidemic and progress in the diagnosis, treatment, prevention, and control of TB [10, 11]. The estimated targets for 2030 are 90% detection and treatment of TB cases, and a cure rate of 90% of detected TB cases [12].

Ethiopia is one of the highest TB burden countries in the world, with an annual incidence of 119 cases per 100,000 populations in 2021 [13]. According to the Ministry of Health (MOH) of Ethiopia, TB is the third leading cause of hospital admissions and the second leading cause of death after malaria [14]. TB case detection, diagnosis, and linkage to TB treatment are the key intervention areas of the TB prevention and control program in Ethiopia [13]. According to Alene et al., the overall pooled prevalence of TB in Ethiopia was 0.19% [15]. The pooled prevalence of any anti-TB drug resistance was reported to be 14.25% [16].

TB is still recognized as a major public health problem in Ethiopia [17]. The potential reasons for this situation in Ethiopia may include inadequate health promotion efforts, insufficient public service regulations, overcrowded housing conditions, and the need for improved awareness campaigns and adherence to anti-TB medications [18, 19]. Particularly in the eastern part of Ethiopia, there needs to be more information and suboptimal effort in early detection, treatment, prevention, and control of TB cases. Therefore, this study was conducted to determine the prevalence of TB, rifampicin-resistant *M. tuberculosis,* and factors associated with TB among patients attending Adama Hospital Medical College (AHMC), Oromia Regional State, Ethiopia.

## 2. Methods

### 2.1. Study Setting and Design

A prospective cross-sectional study was conducted from January 10, 2023, to November 10, 2023, at AHMC in Adama City. Adama is located about 99 km due southeast of Addis Ababa, the capital city of Ethiopia. It is located at a latitude of 8° 54′ north and a longitude of 39° 27′ east. AHMC serves as a referral hospital for the people residing in the East Shewa Zone of the Oromia Regional State and adjacent regions.

### 2.2. Study Population

The study population includes all patients having presumptive pulmonary TB and those who attended AHMC during the study period. Patients with the clinical manifestations of pulmonary TB, such as cough for two or more weeks, chest pain when breathing or coughing, night sweats, weight loss, fatigue, fever, and chills, were included. The sample size was determined using a single population proportion formula, assuming a prevalence of PTB of 50%, a 95% confidence interval, a 5% margin of error, and a 10% nonresponse rate. The minimum sample size was 422. Participants were recruited prospectively in a consecutive manner until the estimated sample size of 422 was obtained.

### 2.3. Data Collection

The questionnaire was prepared after reviewing the related literature [11, 20–24]. Initially, it was prepared in English and then translated into Afaan Oromo and retranslated back into English by a language expert to facilitate respondent understanding, remove sensitive questions, and limit bias during data collection. Trained nurses collected sociodemographic and clinical characteristics from TB-suspected patients using an interviewer-based modality.

### 2.4. Specimen Collection and Processing

A trained microbiologist at the AHMC instructed the study participants to bring two 3–5 mL sputum in 30 min intervals in a screw-cap falcon tube. Physicians or trained nurses collected expectorate sputum and gastric lavage samples from under-five children who could not provide sputum and immediately sent them to the microbiology laboratory at AHMC. The diagnosis of TB was made using Ziehl–Neelsen (ZN) staining technique and the GeneXpert-MTB/RIF assay (Cepheid, CA, USA) [25, 26]. In brief, for ZN, a portion of sputum was placed on a grease-free frosted glass slide using an applicator stick, spread evenly, dried, and heat-fixed. ZN staining was performed according to the SOP of the ZN staining method [27]. For GeneXpert, 2 mL of GeneXpert MTB/RIF specimen reagent buffer was added to 1 mL of sputum specimen using a sterile pipette. The closed specimen container was manually agitated twice for 15 s and allowed to stand at room temperature for 10 min, vortexed after 10 min, and allowed to stand for 5 min, then 2 mL of the inactivated material was transferred to the test cartilage, and the cartilage was then loaded into the GeneXpert device. Finally, the results were interpreted by the GeneXpert diagnostic system from the measured fluorescence signals and automatically displayed after 2 h [25, 26].

### 2.5. Data Management and Quality Control

To ensure the quality and consistency of the questionnaire, a pretest was conducted on 5% of the total sample size (*n* = 21) at Geda Health Center, which is found in Adam City. Two day of training was provided to data collectors and laboratory investigators. Completeness and logical consistency were checked during data collection. Laboratory procedures were performed using the GeneXpert assay and ZN standard operating procedure (SOP) [26]. Positive control and negative control were used from both the GeneXpert assay and the ZN staining method. All reagents were checked before use.

### 2.6. Data Processing and Analysis

Data were visually inspected, coded, and entered into the SPSS Version 20 computer software for analysis. Descriptive statistics and cross-tabulations were used to present the findings. Bivariate logistic regression was performed, and then variables with *p* < 0.25 were further analyzed using multivariable logistic regression. Variables with *p* < 0.05 were interpreted as statistically significant. Sensitivity, specificity, positive predictive value (PPV), negative predictive value (NPV), and likelihood ratio were determined for the ZN staining technique considering GeneXpert as a reference test. The test agreement and the reference method were evaluated using the Kappa value. The Kappa values 0–0.2, 0.21–0.39, 0.4–0.59, 0.6–0.79, 0.8–0.9, and > 0.9 were interpreted as no agreement, minimal agreement, weak agreement, moderate agreement, strong agreement, and almost perfect agreement, respectively [28].

### 2.7. Ethical Approval and Consent to Participate

The study was approved by the Institutional Review Board of AHMC (reference number: AHMC/lab/4/01/2023). The purpose and procedure of the study were explained to the study participants. Data were collected after written informed consent and permission were obtained from participants. Strict confidentiality was maintained using codes only throughout the study.

## 3. Results

### 3.1. Sociodemographic and Clinical Characteristics

In the present study, 424 suspected pulmonary TB patients were included. Of the total participants, 204 (48.1%) were males, and 184 (43.4%) were urban dwellers (Table 1). The mean age of study participants was 32.13, with a minimum age of 4 years and a maximum of 79 years. The mean monthly income of participants was 1213.04 Ethiopian Birr (ETB). The minimum monthly income was 0, with a maximum income of 9500.00 ETB. The minimum family size was 1, and the maximum was 10, with the average family size of 4.72. All participants had a chronic cough, productive sputum, weight loss, night sweats, fatigue, shortness of breath, loss of appetite, and chest pain. About 12% of study participants had a history of contact with TB patients, while 10 of them were HIV-positive (Table 2).

### 3.2. Prevalence and Factors Associated With TB

The prevalence of the ZN staining method- and GeneXpert-confirmed TB was 160 (37.7%), 95% CI: 33–42.7); and 189 (44.6%), 95% CI: 39.8–49.5), respectively. Out of the total study patients who participated in the study, 9 (2.1%) were infected with rifampicin-resistant MTB. Of the 189 confirmed cases of TB, 4.7% were rifampicin-resistant.

The sensitivity, specificity, PPV, and NPV of the ZN staining method in diagnosing TB were 79%, 93.3%, 91.9%, and 84%, respectively (Table 3). The likelihood ratio was 15.6. Kappa value (0.73) indicates the moderate agreement between the ZN staining technique and GeneXpert.

In bivariate logistic regression analysis, gender—male (COR = 1.41, 95% CI [0.96–2.08], *p*=0.077), educational status—no-formal education (COR = 1.80, 95% CI [1.06–3.06], *p*=0.029), educational status—grade 1–8 (COR = 1.72, 95% CI [1.02–2.92], *p*=0.044), history of contact with TB patients (COR = 15.22, 95% CI [5.91–39.18], *p* < 0.001), smoking cigarette (COR = 39.0, 95% CI [5.24–289], *p* < 0.001), and drinking alcohol (COR = 3.98, 95% CI [2.34–6.76], *p* < 0.001) were significantly associated with pulmonary TB. However, only gender—male (AOR = 1.47, 95% CI [0.95−2.26], *p*=0.081), history of contact with TB patient (AOR = 7.19, 95% CI [2.55–20.25], *p* < 0.001), previously treated TB patients (AOR = 3.11, 95% CI [1.49–6.50], *p*=0.003), and smoking cigarette (AOR = 14.8, 95% CI [1.88–117], *p*=0.010) were significantly associated with occurrence of pulmonary TB in multivariable analysis (Tables 4, 5).

## 4. Discussion

The prevention and control of TB are further complicated in resource-limited countries because of the widespread drug-resistant TB, war, poor infrastructure, and fragile health systems. In this study, as detected by GeneXpert we have found a high prevalence (44.6%) of pulmonary TB. Like our study, only two studies from Pakistan reported *a* > 40% prevalence of pulmonary TB [29, 30].

In most cases, our finding is consistently higher than pulmonary TB reported from other countries, including Ethiopia, where the prevalence ranges from 9.3% to 24.3% [22, 24, 31–41]. In other regions of Ethiopia, the prevalence generally remains below 30%, except for Hawassa, Ethiopia, which is recorded at 30.5% [23]. The lowest prevalence is found in Northern Ethiopia at 8.4% [40]. The high prevalence of pulmonary TB found at AHMC should alert concerned bodies and find out the reason behind the high prevalence. We have noted that some African countries have reported an even higher prevalence of pulmonary TB than we have found: Togo, 57% [42], and Central African Republic, 79.1% [43]. The variation in the findings could be due to the nature of the study participants, underlying conditions, study period, and TB control and prevention practices. Countries or parts of the country where there is a high prevalence of pulmonary TB may have poor or below-optimum prevention and control systems.

Among the confirmed TB cases in this study, rifampicin-resistant positive *M. tuberculosis* was 4.7%, which is comparable with reports from Mizan, Ethiopia (5.7%) [20]; Afar, Ethiopia (4.3%) [30]; Hawassa, Ethiopia (4.1%) [23]; Kenya (4.6%) [44]; and Nigeria (6.8%) [45]. Majority of rifampicin-resistant *M. tuberculosis* was detected in patients with no history of TB. There was only a participant with a history of TB who was positive for rifampicin-resistant *M. tuberculosis.* The proportion of rifampicin resistance in this study was notably lower compared to Western Oromia, Ethiopia (25.9%) [35]; East Gojjam Zone, Ethiopia (23.1%) [46]; Enat Hospital, Central Ethiopia (15.8%) [47]; Addis Ababa, Ethiopia (11.11%) [48]; Debre Markos Referral Hospital, Ethiopia (10.3%) [33]; and a systematic review and meta-analysis conducted in Ethiopia (9.67%) [49]. In contrast to the current study, the prevalence of rifampicin-resistant *M. tuberculosis* was even lower in the survey conducted at Jima University Medical Center (3.4%) [38] and in the Federal Medical Center Birnin Kudu, Jigawa State (1.1%) [50]. The data reveal a significant disparity in the occurrence of rifampicin-resistant *M. tuberculosis* in Ethiopia, with the lowest rate documented in Jimma at 3.4% [38] and the highest in Western Oromia at 25.9% [35].

Numerous studies [22, 24, 30, 33, 51] have supported our findings that males are more susceptible to pulmonary TB than females. This discrepancy in risk can be attributed to the higher likelihood of men being exposed to crowded and congested environments during various activities, which increases their vulnerability to the disease.

Patients with a history of contact with TB patients and a previously treated TB were found to have a higher likelihood of being infected with TB compared to those who had no contact with TB patients and no previous history of TB infection. This finding is consistent with findings from other studies [24, 52, 53] and suggests that the mode of TB transmission and reactivation of *M. tuberculosis* bacteria play a role in the increased risk of infection. It is evident that individuals who have had contact with TB patients are at a higher risk of TB infection. This finding underscores the potential impact of increased awareness and education on the ways TB is transmitted. As a significant number of participants in the study area have been in contact with TB patients, addressing this lack of awareness could significantly contribute to TB prevention efforts in the study area.

The current investigation revealed that individuals who smoke cigarettes were 13.21 times more prone to contracting TB compared to nonsmokers (AOR: 14.8 [1.88–117]; *p*=0.010), aligning with findings from previous research studies [22, 24]. The advancement of TB has been associated with a compromised immune system [54–56] due to the detrimental effects of cigarette smoking on the cilia, lungs, and overall defense mechanisms of the body, rendering individuals more vulnerable to *M. tuberculosis* infection.

In Ethiopia, the ZN staining technique is commonly used, and in this study, we assessed its performance by comparing it to the GeneXpert method. GeneXpert detected 29 cases, in which ZN staining had been missed, which aligns with previous research on the performance of GeneXpert and AFB microscopy [57, 58]. The study revealed that the ZN staining technique had an overall sensitivity of 79% and specificity of 93.3%, indicating its effectiveness in diagnosing TB. Interestingly, our findings showed higher sensitivity and specificity than a study conducted in Nigeria, where the sensitivity was 50.0% and specificity was 80.89% [57]. Furthermore, a study from Thailand reported a lower sensitivity of 60.5% but a comparable specificity of 98.5% for the ZN staining technique. Our study's results suggest that the ZN staining technique's performance in Ethiopia is relatively better than in other regions, highlighting its importance in TB diagnosis [59].

### 4.1. Limitation of the Study

As we have used a convenient sampling technique, selection bias could not be avoided. The burden of TB could fluctuate throughout various seasons; nevertheless, our research was not intended to identify the prevalence of TB during different seasons.

## 5. Conclusion

In this study, it was found that the occurrence of pulmonary TB was relatively high, while the prevalence of rifampicin-resistant *M. tuberculosis* was at a moderate level. The study revealed that individuals who were male, had a history of contact with TB patients, had previous TB infections, and were smokers, were more susceptible to developing TB infection. Despite the national program aiming to eradicate TB by 2030, the prevalence of TB remained high in the study area. Therefore, health programmers and stakeholders must intensify their efforts in implementing the proposed strategy of early case detection and management, and implementing appropriate control and prevention methods.

## Figures and Tables

**Table 1:** Sociodemographic characteristics of TB-suspected patients attending Adama Hospital Medical College from January 1, 2023, to November 20, 2023 (*N* = 424).

Variables	Category	Frequency (%)	GeneXpert diagnosis, *n* (%)		*X* ^2^	*p* value
			Positive	Negative		
Gender	Male	204 (48.1)	100 (49)	104 (51)	3.14	0.08
	Female	220 (51.9)	89 (40.5)	131 (59.5)		

Residence	Urban	184 (43.4)	83 (45.1)	101 (54.9)	0.037	0.85
	Rural	240 (56.6)	106 (44.2)	134 (55.8)		

Marital status	Single	164 (38.67)	71 (43.3)	93 (56.7)	848	0.47
	Married	253 (59.67)	114 (45.1)	139 (54.9)		
	Widowed	7 (1.65)	4 (57.1)	3 (42.9)		

Educational status	No formal education	127 (29.95)	62 (48.8)	65 (51.2)	1272	0.47
	Grade 1–6	128 (30.19	61 (47.7)	67 (52.3)		
	Grade 9–12	62 (14.62)	29 (46.8)	33 (53.2)		
	Diploma or degree	107 (25.23)	37 (34.6)	70 (65.4)		

Occupation	Farmer	97 (22.9)	49 (50.5)	48 (49.5)	2120	0.46
	Student	153 (36.1)	67 (43.8)	86 (56.2)		
	Housewife	79 (18.6)	39 (49.4)	40 (50.6)		
	Employee	76 (17.9)	26 (34.2)	50 (65.8)		
	Driver	6 (1.4)	3 (50)	3 (50)		
	Others	13 (3.1)	5 (38.5)	8 (61.5)		

Number of rooms in a house	One	9 (2.1)	4 (44.4)	5 (55.6)	17.25	−0.004
	Two	134 (31.6)	74 (55.2)	60 (44.8)		
	Three	110 (25.9)	54 (49.1)	56 (50.9)		
	Four	102 (24.1)	33 (32.4)	69 (67.6)		
	Five	54 (12.7)	17 (31.5)	37 (68.5)		
	Six	15 (3.5)	7 (46.7)	8 (53.3)		

Number of windows in a house	One	48 (11.3)	21 (43.8)	27 (56.2)	8.46	0.076
	Two	237 (55.9)	119 (50.2)	118 (49.8)		
	Three	80 (18.9)	28 (35)	52 (65)		
	Four	48 (11.3)	18 (37.5)	30 (62.5)		
	Five	11 (2.6)	3 (72.7)	8 (22.7)		

**Table 2:** Clinical characteristics of TB-suspected patients attending Adama Hospital Medical College from January 1, 2023, to November 20, 2023 (*N* = 424).

Variables	Category	Frequency (%)	GeneXpert diagnosis, *n* (%)		*X* ^2^	*p* value
			Positive	Negative		
History of contact with TB patient	Yes	52 (12.3)	47 (90.4)	5 (9.6)	50.34	< 0.0001
	No	372 (87.7)	142 (38.2)	23 (61.8)		

Congested transport	Yes	287 (67.7)	136 (47.4)	151 (52.6)	2.84	0.092
	No	137 (32.3)	53 (38.7)	84 (61.3)		

History of imprisonment	Yes	33 (7.8)	27 (81.8)	6 (18.2)	20	< 0.0001
	No	391 (92.2)	162 (41.4)	229 (58.6)		

History of infection with TB	Yes	77 (18.2)	63 (81.8)	14 (18.2)	52.8	< 0.0001
	No	347 (81.8)	126 (36.3)	221 (63.7)		

HIV status	No	393 (92.7)	159 (40.5)	234 (59.5)	36.9	< 0.0001
	Yes	10 (2.4)	10 (100)	0 (0)		
	Unknown	21 (2.4)	20 (95.2)	1 (4.8)		

Smoking cigarette	Yes	28 (6.6)	27 (96.4)	1 (3.6)	32.6	< 0.0001
	No	396 (92.5)	162 (40.9)	234 (59.1)		

Frequency of smoking	Every day	24 (85.7)	23 (95.8)	1 (4.2)	0.314	0.56
	Per week	4 (50)	4 (100)	0 (0)		

Drinking alcohol	Yes	80 (18.9)	57 (71.2)	23 (28.8)	28.4	< 0.0001
	No	344 (81.1)	132 (38.4)	212 (61.6)		

Frequency of alcohol drinking	Every day	22 (27.5)	19 (86.4)	3 (13.6)	4.42	0.11
	Per week	52 (65)	35 (67.3)	17 (32.7)		
	Per month	6 (7.5)	3 (50)	3 (50)		

Duration of feeling signs and symptoms of TB	< 2 weeks	101 (23.8)	47 (46.5)	54 (53.5)	0.2	0.65
	2–5 weeks	323 (76.2)	142 (44)	181 (56)		

Drinking of raw milk	Yes	396 (93.4)	178 (44.9)	218 (55.1)	0.34	0.56
	No	28 (6.6)	11 (39.3)	17 (60.7)		

**Table 3:** Performance of Zn staining in diagnosing pulmonary tuberculosis.

		GeneXpert		
		Detected	Not detected	Total
ZN staining in the diagnosis of PTB	Positive	147	13	160
	Negative	42	222	264
	Total	186	238	424

*Note:* Sensitivity = 147/186 × 100 = 79%. Specificity = 222/238 × 100 = 93.3%. PVP = 147/160 × 100 = 91.9%. NPV = 222/264 × 100 = 84%.

**Table 4:** Bivariable analysis of sociodemographic and clinical factors among TB-suspected patients in Adama Hospital Medical College, 2023 (*N* = 424).

Variable		GeneXpert test result *n* (%)		COR (95% CI)	*p* value
		Positive	Negative		
Gender	Male	100 (49)	104 (51)	1.41 (0.96–2.08)	0.077
	Female	89 (40.5)	131 (59.5)	1	

Residence	Urban	83 (45.1)	101 (54.9)	1.04 (0.71–1.53)	0.847
	Rural	106 (44.2)	134 (55.8)	1	

Marital status	Single	71 (43.3)	93 (56.7)	0.57 (0.12–2.64)	0.475
	Married	114 (45.1)	139 (54.9)	0.62 (0.13–2.80)	0.530
	Widowed	4 (57.1)	3 (42.9)	1	

Educational status	No formal education	62 (48.8)	65 (51.2)	1.80 (1.06–3.06)	0.029
	Grade 1–8	61 (47.7)	67 (52.3)	1.72 (1.02–2.92)	0.044
	Grade 9–12	29 (46.8)	33 (53.2)	1.66 (0.88–3.15)	0.119
	Diploma or degree	37 (34.6)	70 (65.4)	1	

Occupational status	Farmer	49 (50.5)	48 (49.5)	1.63 (0.50–5.35)	0.418
	Student	67 (43.8)	86 (56.2)	1.25 (0.39–3.98)	0.710
	Housewife	39 (49.4)	40 (50.6)	1.56 (0.47–5.19)	0.468
	Employee	26 (34.2)	50 (65.8)	0.83 (0.25–2.80)	0.766
	Driver	3 (50)	3 (50)	1.60 (0.23–11.26)	0.637
	Others	5 (38.5)	8 (61.5)	1	

History of contact with TB patient	Yes	47 (90.4)	5 (9.6)	15.22 (5.91–39.18)	< 0.001
	No	142 (38.2)	230 (61.8)	1	

Congested transport	Yes	136 (47.4)	151 (52.6)	1.43 (0.94–2.16)	0.092
	No	53 (38.7)	84 (61.3)	1	

History of imprisonment	Yes	27 (81.8)	6 (18.2)	6.36 (2.57–15.76)	< 0.001
	No	162 (41.4)	229 (58.6)	1	

Previously treated TB patients	Yes	63 (81.8)	14 (18.2)	7.89 (4.25–14.66)	< 0.001
	No	126 (36.3)	221 (63.7)	1	

Smoking of cigarette	Yes	27 (96.4)	1 (3.6)	39.0 (5.24–289)	< 0.001
	No	162 (40.9)	234 (59.1)	1	

Drinking of alcohol	Yes	57 (71.2)	23 (28.8)	3.98 (2.34–6.76)	< 0.001
	No	132 (38.4)	212 (61.6)	1	

Drinking of raw milk	Yes	178 (44.9)	218 (55.1)	1.26 (0.58–2.76)	0.560
	No	11 (39.3)	17 (60.7)	1	

**Table 5:** Multivariable analysis of contributing factors among TB-suspected patients in Adama Hospital Medical College, 2023 (*N* = 424).

Variables		GeneXpert test result *n* (%)		COR (95% CI)	*p* value	AOR (95% CI)	*p* value
		Positive	Negative				
History of contact with TB patient	Yes	47 (90.4)	5 (9.6)	15.22 (5.91–39.18)	< 0.001	7.19 (2.55–20.25)	< 0.001
	No	142 (38.2)	230 (61.8)	1			

Congested transport	Yes	136 (47.4)	151 (52.6)	1.43 (0.94–2.16)	0.092		
	No	53 (38.7)	84 (61.3)	1			

History of imprisonment	Yes	27 (81.8)	6 (18.2)	6.36 (2.57–15.76)	< 0.001		
	No	162 (41.4)	229 (58.6)	1			

History of TB infection	Yes	63 (81.8)	14 (18.2)	7.89 (4.25–14.66)	< 0.001	3.11 (1.49–6.50)	0.003
	No	126 (36.3)	221 (63.7)	1			

Smoking of cigarette	Yes	27 (96.4)	1 (3.6)	39.0 (5.24–289.8)	< 0.001	14.8 (1.88–117)	0.010
	No	162 (40.9)	234 (59.1)	1			

Drinking of alcohol	Yes	57 (71.2)	23 (28.8)	3.98 (2.34–6.76)	< 0.001		
	No	132 (38.4)	212 (61.6)	1			

Gender	Male	100 (49)	104 (51)	1.41 (0.96–2.08)	0.077	1.47 (0.95–2.26)	0.081
	Female	89 (40.5)	131 (59.5)	1			

Educational status	No formal education	62 (48.8)	65 (51.2)	1.80 (1.06–3.06)	0.029		
	Grade 1–8	61 (47.7)	67 (52.3)	1.72 (1.02–2.92)	0.044		
	Grade 9–12	29 (46.8)	33 (53.2)	1.66 (0.88–3.15)	0.119		
	Diploma or degree	37 (34.6)	70 (65.4)	1			

Abbreviations: ⁣^*∗*^AOR, adjusted odds ratio; ⁣^*∗*^COR, crude odds ratio.

## Data Availability

Datasets used for the analysis can be obtained from the corresponding author.

## Nomenclature

TB: Tuberculosis
WHO: World Health Organization
SOP: Standard operating procedure
AHMC: Adama Hospital Medical College

## References

[1] Khan M., Raja Z., Ahmed H., Rauf A. (2019). A Pattern of Tuberculosis Infection an Overview. *Pakistan Journal of Biotechnology*.

[2] Cheesbrough M. (1981). Medical Laboratory Manual for Tropical Countries: 14 Bevills Close, Doddington, Cambridgeshire.

[3] Cambier C. J., Falkow S., Ramakrishnan L. (2014). Host Evasion and Exploitation Schemes of Mycobacterium Tuberculosis. *Cell*.

[4] Cheesbrough M. (1984). Medical Laboratory Manual for Tropical Countries/Vol.ii, Microbiology. Doddington (14 Bevills Close, Doddington, Cambs. Pe15 0tt): M. Cheesbrough Doddington.

[5] WHO (2019). *Global Status Report on Alcohol and Health 2018*.

[6] WHO Global Tuberculosis Report 2024. https://www.who.int/teams/global-tuberculosis-programme/tb-reports/global-tuberculosis-report-2024.

[7] Zaman K. (2010). Tuberculosis: A Global Health Problem. *Journal of Health, Population and Nutrition*.

[8] Korenromp E. L., Glaziou P., Fitzpatrick C. (2012). Implementing the Global Plan to Stop TB, 2011–2015-Optimizing Allocations and the Global Fund’s Contribution: A Scenario Projections Study. *PLoS One*.

[9] WHO (2022). *Practical Manual on Tuberculosis Laboratory Strengthening: 2022 Update*.

[10] Christof C., Nussbaumer-Streit B., Gartlehner G. (2020). WHO Guidelines on Tuberculosis Infection Prevention and Control. *Das Gesundheitswesen*.

[11] Uplekar M., Weil D., Lonnroth K. (2015). WHO’s New End TB Strategy. *The Lancet*.

[12] MOH (2022). *Annual Performance, Report, 2014 EFY/2021–2022*.

[13] FMOH (2017). DR-TB and Leprosy in Ethiopia.

[14] Alene K. A., Python A., Weiss D. J. (2023). Mapping Tuberculosis Prevalence in Ethiopia Using Geospatial Meta-Analysis. *International Journal of Epidemiology*.

[15] Reta M. A., Tamene B. A., Abate B. B., Mensah E., Maningi N. E., Fourie P. B. (2022). Mycobacterium Tuberculosis Drug Resistance in Ethiopia: An Updated Systematic Review and Meta-Analysis. *Tropical Medicine and Infectious Disease*.

[16] Faisal S. (2023). Global Tuberculosis Epidemiology. *Tuberculosis: Integrated Studies for a Complex Disease*.

[17] Kuupiel D., Vezi P., Bawontuo V., Osei E., Mashamba-Thompson T. P. (2020). Tuberculosis Active Case-Finding Interventions and Approaches for Prisoners in Sub-Saharan Africa: A Systematic Scoping Review. *Bio Medical Central Infectious Diseases*.

[18] Wasihun A. G., Hailu G. G., Dejene T. A. (2021). Prevalence of Mycobacterium Tuberculosis (Rifampicin-Resistant MTB) and Associated Risk Actors Among Pulmonary Presumptive TB Patients in Eastern Amhara, Ethiopia: 2015–2019. *Infectious Disease and Therapy*.

[19] Berhe G., Enquselassie F., Aseffa A. (2013). Assessment of Risk Factors for Development of Active Pulmonary Tuberculosis in Northern Part of Ethiopia: A Matched Case Control Study. *Ethiopian Medical Journal*.

[20] Dejene T. A., Hailu G. G., Kahsay A. G., Wasihun A. G. (2023). Pulmonary Tuberculosis and Rifampicin Resistant Mycobacterium Tuberculosis in Children and Adolescents Using Gene Xpert MTB/RIF Assay in Tigray, Northern Ethiopia. *Infection and Drug Resistance*.

[21] Abayneh M., Teressa M. (2022). Detection of Mycobacterium Tuberculosis Using Gene Xpert-Mtb/Rif Assay Among Tuberculosis Suspected Patients at Mizan-Tepi University Teaching Hospital, Southwest Ethiopia: An Institution Based Cross-Sectional Study. *PLoS One*.

[22] Andarge D. B., Anticho T. L., Jara G. M., Ali M. M. (2021). Prevalence of Mycobacterium Tuberculosis Infection and Rifampicin Resistance Among Presumptive Tuberculosis Cases Visiting Tuberculosis Clinic of Adare General Hospital, Southern Ethiopia. *SAGE Open Medicine*.

[23] Diriba K., Churiso G. (2022). The Prevalence of Mycobacterium Tuberculosis Using Gene Xpert Among Tuberculosis Suspected Patients in Gedeo Zone, Southern Ethiopia. *European Journal of Medical Research*.

[24] Solovic I., Abubakar I., Sotgiu G. (2017). Standard Operating Procedures for Tuberculosis Care. *European Respiratory Journal*.

[25] Kim M. J., Nam Y. S., Cho S. Y., Park T. S., Lee H. J. (2015). Comparison of the Xpert MTB/RIF Assay and Real-Time PCR for the Detection of Mycobacterium Tuberculosis. *Annals of Clinical and Laboratory Science*.

[26] Bhirud P., Joshi A., Hirani N., Chowdhary A. (2017). Rapid Laboratory Diagnosis of Pulmonary Tuberculosis. *International Journal of Mycobacteriology*.

[27] McHugh M. L. (2012). Interrater Reliability: The Kappa Statistic. *Biochemia Medica*.

[28] Iram S., Zeenat A., Hussain S., Wasim Yusuf N., Aslam M. (2015). Rapid Diagnosis of Tuberculosis Using Xpert MTB/RIF Assay-Report From a Developing Country. *Pakistan Journal of Medical Sciences*.

[29] Saeed M. (2020). GeneXpert: A New Tool for the Rapid Detection of Rifampicin Resistance in mycobacterium Tuberculosis. *International Journal of Infectious Diseases*.

[30] Adejumo O. A., Olusola-Faleye B., Adepoju V. (2018). Prevalence of Rifampicin Resistant Tuberculosis and Associated Factors Among Presumptive Tuberculosis Patients in a Secondary Referral Hospital in Lagos Nigeria. *African Health Sciences*.

[31] Abay G. K., Abraha B. H. (2020). Trends of Mycobacterium Tuberculosis and Rifampicin Resistance in Adigrat General Hospital, Eastern Zone of Tigrai, North Ethiopia. *Tropical Diseases, Travel Medicine and Vaccines*.

[32] Mulu W., Abera B., Yimer M., Hailu T., Ayele H., Abate D. (2017). Rifampicin-resistance Pattern of Mycobacterium Tuberculosis and Associated Factors Among Presumptive Tuberculosis Patients Referred to Debre Markos Referral Hospital, Ethiopia: A Cross-Sectional Study. *Bio Medical Central Research Notes*.

[33] Khadka P., Thapaliya J., Basnet R. B., Ghimire G. R., Amatya J., Rijal B. P. (2019). Diagnosis of Tuberculosis From Smear-Negative Presumptive TB Cases Using Xpert MTB/Rif Assay: A Cross-Sectional Study From Nepal. *BMC Infectious Diseases*.

[34] Zewdie O., Dabsu R., Kifle E., Befikadu D. (2020). Rifampicin-Resistant Multidrug-Resistant Tuberculosis Cases in Selected Hospitals in Western Oromia, Ethiopia: Cross-Sectional Retrospective Study. *Infection and Drug Resistance*.

[35] Ikuabe P. O., Ebuenyi I. D. (2018). Prevalence of Rifampicin Resistance By Automated Genexpert Rifampicin Assay in Patients With Pulmonary Tuberculosis in Yenagoa, Nigeria. *The Pan African medical journal*.

[36] Mechal Y., Benaissa E., El Mrimar N. (2019). Evaluation of GeneXpert MTB/RIF System Performances in the Diagnosis of Extrapulmonary Tuberculosis. *Bio Medical Central Infectious Diseases*.

[37] Admassu W., Ayelign B., Abebe G., Tadesse M. (2022). Detection of Mycobacterium Tuberculosis and Rifampicin Resistance by Xpert MTB/RIF Assay Among Presumptive Tuberculosis Cases at Jimma University Medical Center, Southwest Ethiopia. *PLoS One*.

[38] Gebretsadik D., Ahmed N., Kebede E., Mohammed M., Belete M. A. (2020). Prevalence of Tuberculosis by Automated GeneXpert Rifampicin Assay and Associated Risk Factors Among Presumptive Pulmonary Tuberculosis Patients at Ataye District Hospital, North East Ethiopia. *Infection and Drug Resistance*.

[39] Demissie T. A., Belayneh D. (2021). Magnitude of Mycobacterium Tuberculosis Infection and its Resistance to Rifampicin Using Xpert-MTB/RIF Assay Among Presumptive Tuberculosis Patients at Motta General Hospital, Northwest Ethiopia. *Infection and Drug Resistance*.

[40] Mohammed H., Oljira L., Roba K. T. (2021). Tuberculosis Prevalence and Predictors Among Health Care-Seeking People Screened for Cough of Any Duration in Ethiopia: A Multicenter Cross-Sectional Study. *Frontiers in Public Health*.

[41] Dagnra A. Y., Mlaga K. D., Adjoh K., Kadanga E., Disse K., Adekambi T. (2015). Prevalence of Multidrug-Resistant Tuberculosis Cases Among HIV-Positive and HIV-Negative Patients Eligible for Retreatment Regimen in Togo Using GeneXpert MTB/RIF. *New microbes and new infections*.

[42] Farra A., Manirakiza A., Yambiyo B. M. (2019). Surveillance of Rifampicin Resistance With GeneXpert MTB/RIF in the National Reference Laboratory for Tuberculosis at the Institut Pasteur in Bangui, 2015–2017. *Open Forum Infectious Diseases*.

[43] Cattaneo P., Mulongo C. M., Morino G. (2023). Burden of Pulmonary Rifampicin-Resistant Tuberculosis in Kajiado, Kenya: An Observational Study. *Microorganisms*.

[44] Ulasi A., Nwachukwu N., Onyeagba R., Umeham S., Amadi A. (2022). Prevalence of Rifampicin Resistant Tuberculosis Among Pulmonary Tuberculosis Patients in Enugu, Nigeria. *African Health Sciences*.

[45] Toru M., Baye A., Gebeyehu Z., Abebaw A., Reta A. (2022). Prevalence, Associated Factors and Rifampicin Resistance Pattern of Pulmonary Tuberculosis Among HIV-Positive Patients Attending Antiretroviral Treatment Clinic at East Gojjam Zone, Ethiopia: An Institution-Based Cross-Sectional Study. *Journal of Clinical Tuberculosis and Other Mycobacterial Diseases*.

[46] Selfegna S., Alelign A. (2022). Detection of Mycobacterium Tuberculosis and Rifampicin Resistance Using GeneXpert MTB/RIF Assay at Enat Hospital, Central Ethiopia. *Tuberculosis research and treatment*.

[47] Akalu G. T., Tessema B., Petros B. (2022). High Proportion of RR-TB and Mutations Conferring RR outside of the RRDR of the RpoB Gene Detected in GeneXpert MTB/RIF Assay Positive Pulmonary Tuberculosis Cases, in Addis Ababa, Ethiopia. *PLoS One*.

[48] Demelash M., Nibret E., Hailegebriel T., Minichil Z., Mekonnen D. (2023). Prevalence of Rifampicin Resistant Pulmonary Tuberculosis Using GeneXpert Assay in Ethiopia, A Systematic Review and Meta-Analysis. *Heliyon*.

[49] Olalere O. S., Olley M., Clement O. O., Paul I. I. (2022). Prevalence of M. Tuberculosis and Associated Risk Factors Among Suspected Patients in Federal Medical Centre Birnin Kudu, Jigawa State. *Sokoto Journal of Medical Laboratory Science.*.

[50] Snow K., Yadav R., Denholm D., Sawyer S., Graham S. (2018). Tuberculosis Among Children, Adolescents and Young Adults in the Philippines: A Surveillance Report. *Western Pacific Surveillance and Response Journal*.

[51] Trunz B. B., Fine P., Dye C. (2006). Effect of BCG Vaccination on Childhood Tuberculous Meningitis and Miliary Tuberculosis Worldwide: A Meta-Analysis and Assessment of Cost-Effectiveness. *The Lancet*.

[52] Reichler M. R., Reves R., Bur S. (2002). Evaluation of Investigations Conducted to Detect and Prevent Transmission of Tuberculosis. *Journal of the American Medical Association*.

[53] Zuliartha N., Daulay R. M., Deliana M., Dalimunthe W., Daulay R. S. (2015). Association Between Passive Smoking and Mycobacterium Tuberculosis Infection in Children With Household TB Contact. *Paediatrica Indonesiana*.

[54] Collins P. Y., Insel T. R., Chockalingam A., Daar A., Maddox Y. T. (2013). Grand Challenges in Global Mental Health: Integration in Research, Policy, and Practice. *PLoS Medicine*.

[55] Altet M. N., Alcaide J., Plans P. (1996). Passive Smoking and Risk of Pulmonary Tuberculosis in Children Immediately Following Infection. A Case-Control Study. *Tubercle and Lung Disease*.

[56] Ejeh E. F., Undiandeye A., Akinseye V. O. (2018). Diagnostic Performance of GeneXpert and Ziehl-Neelson Microscopy in the Detection of Tuberculosis in Benue State, Nigeria. *Alexandria Journal of Medicine*.

[57] Opota O., Senn L., Prod’hom G. (2016). Added Value of Molecular Assay Xpert MTB/RIF Compared to Sputum Smear Microscopy to Assess the Risk of Tuberculosis Transmission in a Low-Prevalence Country. *Clinical Microbiology and Infection*.

[58] Pinyopornpanish K., Chaiwarith R., Pantip C. (2015). Comparison of Xpert MTB/RIF Assay and the Conventional Sputum Microscopy in Detecting Mycobacterium Tuberculosis in Northern Thailand. *Tuberculosis Research and Treatment*.

[59] Gebrehiwet G. B., Kahsay A. G., Welekidan L. N., Hagos A. K., Abay G. K., Hagos D. G. (2019). Rifampicin Resistant Tuberculosis in Presumptive Pulmonary Tuberculosis Cases in Dubti Hospital, Afar, Ethiopia. *The Journal of Infection in Developing Countries*.

